# 1-(4-*tert*-Butyl­benz­yl)pyrimidine-2,4(1*H*,3*H*)-dione

**DOI:** 10.1107/S1600536812008999

**Published:** 2012-03-14

**Authors:** Hong-Sheng Wang, Gong-Chun Li

**Affiliations:** a School of Chemistry and Chemical Engineering, Xuchang University, Xuchang, Henan Province 461000, People’s Republic of China

## Abstract

The asymmetric unit of the title compound, C_15_H_18_N_2_O_2_, contains two independent mol­ecules with essentially identical geometries and conformations. The dihedral angles between the benzene and pyrimidine rings in the two mol­ecules are 89.96 (11) and 73.91 (11)°. The six methyl groups are disordered over two sets of sites, with site occupancies of 0.545 (4):0.455 (4) and 0.542 (7):0.458 (7) in the two mol­ecules. The crystal structure is stabilized by N—H⋯O hydrogen bonds.

## Related literature

For the bioactivity of pyrimidine-2,4(1*H*,3*H*)-diones, see: Konz (1997 ▶); Reinhard *et al.* (2004 ▶); Komori & Sanemitsu (2002 ▶); Radatus & Karimian (1993 ▶); Starrett *et al.* (1992 ▶). For a related structure, see: Li *et al.* (2005 ▶).
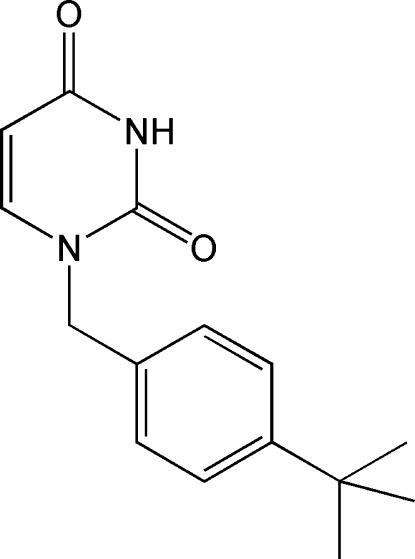


## Experimental

### 

#### Crystal data

C_15_H_18_N_2_O_2_
*M* *_r_* = 258.31
Monoclinic, 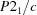

*a* = 20.853 (7) Å
*b* = 10.013 (4) Å
*c* = 13.893 (5) Å
β = 94.915 (6)°
*V* = 2890.2 (18) Å^3^
*Z* = 8
Mo *K*α radiation
μ = 0.08 mm^−1^
*T* = 294 K
0.40 × 0.28 × 0.20 mm

#### Data collection

Bruker SMART CCD area-detector diffractometer
Absorption correction: multi-scan (*SADABS*; Bruker, 1999 ▶) *T*_min_ = 0.969, *T*_max_ = 0.984
14804 measured reflections
5292 independent reflections
2946 reflections with *I* > 2σ(*I*)
*R*_int_ = 0.042

#### Refinement

*R*[*F* ^2^ > 2σ(*F* ^2^)] = 0.063
*wR*(*F* ^2^) = 0.207
*S* = 1.02
5292 reflections
351 parameters
186 restraints
H-atom parameters constrained
Δρ_max_ = 0.50 e Å^−3^
Δρ_min_ = −0.31 e Å^−3^

### 

Data collection: *SMART* (Bruker, 1999 ▶); cell refinement: *SAINT* (Bruker, 1999 ▶); data reduction: *SAINT*; program(s) used to solve structure: *SHELXS97* (Sheldrick, 2008 ▶); program(s) used to refine structure: *SHELXL97* (Sheldrick, 2008 ▶); molecular graphics: *SHELXTL* (Sheldrick, 2008 ▶); software used to prepare material for publication: *SHELXTL*.

## Supplementary Material

Crystal structure: contains datablock(s) global, I. DOI: 10.1107/S1600536812008999/fj2505sup1.cif

Structure factors: contains datablock(s) I. DOI: 10.1107/S1600536812008999/fj2505Isup2.hkl

Supplementary material file. DOI: 10.1107/S1600536812008999/fj2505Isup3.cml

Additional supplementary materials:  crystallographic information; 3D view; checkCIF report

## Figures and Tables

**Table 1:** Hydrogen-bond geometry (Å, °)

*D*—H⋯*A*	*D*—H	H⋯*A*	*D*⋯*A*	*D*—H⋯*A*
N1—H1⋯O3^i^	0.86	2.06	2.915 (3)	174
N3—H3⋯O4^ii^	0.86	2.03	2.851 (3)	160

Symmetry codes: (i) 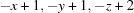; (ii) 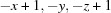.

**Fig. 1.**
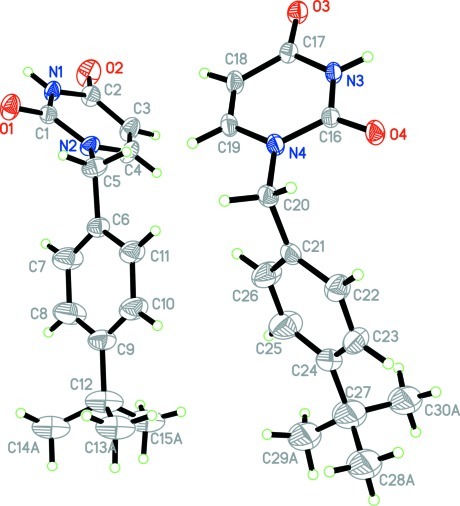
The asymmetric unit of the title compound, (I), with displacement ellipsoids drawn at the 30% probability level.

**Fig. 2.**
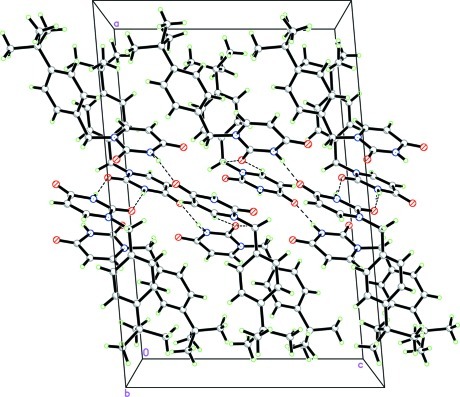
The packing diagram of the title compound. Intermolecular hydrogen bonds are shown as dashed line.

## supplementary crystallographic information

### Comment

Derivatives of pyrimidine-2,4(1*H*,3*H*)-dione are very important molecules in biology and have many application in the areas of herbicide (Konz, 1997; Reinhard *et al.*, 2004; Komori and Sanemitsu 2002). Derivatives of pyrimidine-2,4(1*H*,3*H*)-dione have also been developed as antiviral agents, shch as AZT which is the most widely used anti-AIDS drug (Radatus & Karimian, 1993) and stavudine which is the most widely used anti-HIV drug (Starrett *et al.*, 1992). In order to discover further biologically active pyrimidine compounds, the title compound, (I), was synthesized and its crystal structure determined (Fig. 1).

In the crystal structure of the title molecule, The asymmetric unit contains two independent molecules, with essentially identical geometries and conformations. The dihedral angles between the benzene rings and the pyrimidine rings in the two molecules are 89.96 (0.11) and 73.91 (0.11)°. The six methyl groups are disordered over two positions, with site-occupancies of 0.545 (4):0.455 (4) and 0.542 (7):0.458 (7) in the two molecules. The crystal structure is stabilized by N—H···O hydrogen bonds. For a crystal structure related to the title compound, see: Li *et al.* (2005).

### Experimental

Uracil (0.56 g, 5 mmol) and anhydrous potassium carbonate (0.84 g, 6 mmol) were mixed in *N*,*N*-dimethylformamide (20 ml). A solution of 4-tertbutylbenzyl chloride (0.92 g, 5 mmol) in acetone (10 ml) was then added dropwise, with stirring, at room temperature, and the mixture was stirred for another 10 h and then refluxed for 4 h. The solvent was evaporated *in vacuo* and the residue was washed with water. The resulting white precipitate was filtered off and purified by column chromatography on silica gel (petroleum ether:ethyl acetate = 2:1). The title compound was recrystallized from ethanol and single crystals of (I) were obtained.

### Refinement

All H atoms were placed in calculated positions, with C—H(aromatic) = 0.93 Å and C—H(aliphatic) = 0.96 Å or 0.97 Å, and included in the final cycles of refinement using a riding model, with *U*_iso_(H) = 1.2Ueq(C).

### Crystal data

**Table:**

C_15_H_18_N_2_O_2_	*F*(000) = 1104
*M**_r_* = 258.31	*D*_x_ = 1.187 Mg m^−^^3^
Monoclinic, *P*2_1_/*c*	Mo *K*α radiation, λ = 0.71073 Å
Hall symbol: -P 2ybc	Cell parameters from 3207 reflections
*a* = 20.853 (7) Å	θ = 2.3–22.9°
*b* = 10.013 (4) Å	µ = 0.08 mm^−^^1^
*c* = 13.893 (5) Å	*T* = 294 K
β = 94.915 (6)°	Prism, colourless
*V* = 2890.2 (18) Å^3^	0.40 × 0.28 × 0.20 mm
*Z* = 8	

### Data collection

**Table:**

Bruker SMART CCD area-detector diffractometer	5292 independent reflections
Radiation source: fine-focus sealed tube	2946 reflections with *I* > 2σ(*I*)
Graphite monochromator	*R*_int_ = 0.042
phi and ω scans	θ_max_ = 25.4°, θ_min_ = 1.0°
Absorption correction: multi-scan (*SADABS*; Bruker, 1999)	*h* = −25→22
*T*_min_ = 0.969, *T*_max_ = 0.984	*k* = −10→12
14804 measured reflections	*l* = −16→16

### Refinement

**Table:**

Refinement on *F*^2^	Primary atom site location: structure-invariant direct methods
Least-squares matrix: full	Secondary atom site location: difference Fourier map
*R*[*F*^2^ > 2σ(*F*^2^)] = 0.063	Hydrogen site location: inferred from neighbouring sites
*wR*(*F*^2^) = 0.207	H-atom parameters constrained
*S* = 1.02	*w* = 1/[σ^2^(*F*_o_^2^) + (0.093*P*)^2^ + 1.8764*P*] where *P* = (*F*_o_^2^ + 2*F*_c_^2^)/3
5292 reflections	(Δ/σ)_max_ = 0.016
351 parameters	Δρ_max_ = 0.50 e Å^−^^3^
186 restraints	Δρ_min_ = −0.31 e Å^−^^3^

### Special details

**Table:**

Geometry. All e.s.d.'s (except the e.s.d. in the dihedral angle between two l.s. planes) are estimated using the full covariance matrix. The cell e.s.d.'s are taken into account individually in the estimation of e.s.d.'s in distances, angles and torsion angles; correlations between e.s.d.'s in cell parameters are only used when they are defined by crystal symmetry. An approximate (isotropic) treatment of cell e.s.d.'s is used for estimating e.s.d.'s involving l.s. planes.
Refinement. Refinement of *F*^2^ against ALL reflections. The weighted *R*-factor *wR* and goodness of fit *S* are based on *F*^2^, conventional *R*-factors *R* are based on *F*, with *F* set to zero for negative *F*^2^. The threshold expression of *F*^2^ > σ(*F*^2^) is used only for calculating *R*-factors(gt) *etc*. and is not relevant to the choice of reflections for refinement. *R*-factors based on *F*^2^ are statistically about twice as large as those based on *F*, and *R*- factors based on ALL data will be even larger.

### Fractional atomic coordinates and isotropic or equivalent isotropic displacement parameters (Å^2^)

**Table:**

	*x*	*y*	*z*	*U*_iso_*/*U*_eq_	Occ. (<1)
O1	0.59221 (12)	0.8165 (2)	1.02506 (16)	0.0590 (7)	
O2	0.63012 (14)	0.5542 (3)	1.28734 (17)	0.0779 (8)	
O3	0.47352 (10)	0.14989 (19)	0.74452 (13)	0.0439 (5)	
O4	0.54683 (10)	0.11521 (19)	0.44745 (14)	0.0468 (6)	
N1	0.61343 (12)	0.6826 (2)	1.15484 (16)	0.0424 (6)	
H1	0.5903	0.7343	1.1873	0.051*	
N2	0.65534 (12)	0.6327 (3)	1.00998 (17)	0.0436 (6)	
N3	0.51352 (11)	0.1350 (2)	0.59833 (15)	0.0352 (6)	
H3	0.5018	0.0526	0.5959	0.042*	
N4	0.56191 (11)	0.3135 (2)	0.52653 (16)	0.0358 (6)	
C1	0.61872 (14)	0.7185 (3)	1.0607 (2)	0.0390 (7)	
C2	0.64043 (16)	0.5741 (3)	1.2041 (2)	0.0501 (8)	
C3	0.67929 (17)	0.4932 (3)	1.1461 (2)	0.0593 (9)	
H3A	0.7011	0.4196	1.1732	0.071*	
C4	0.68399 (16)	0.5235 (3)	1.0539 (2)	0.0545 (9)	
H4	0.7080	0.4676	1.0174	0.065*	
C5	0.65428 (15)	0.6492 (4)	0.9050 (2)	0.0528 (9)	
H5A	0.6274	0.7256	0.8861	0.063*	
H5B	0.6343	0.5710	0.8742	0.063*	
C6	0.71906 (16)	0.6689 (3)	0.8674 (2)	0.0481 (8)	
C7	0.7650 (2)	0.7498 (5)	0.9107 (3)	0.0859 (13)	
H7	0.7580	0.7906	0.9690	0.103*	
C8	0.8220 (2)	0.7726 (5)	0.8696 (3)	0.0951 (14)	
H8	0.8522	0.8292	0.9014	0.114*	
C9	0.83593 (18)	0.7163 (4)	0.7849 (3)	0.0681 (10)	
C10	0.78888 (19)	0.6360 (4)	0.7410 (3)	0.0739 (11)	
H10	0.7955	0.5968	0.6820	0.089*	
C11	0.73176 (17)	0.6115 (4)	0.7819 (2)	0.0622 (10)	
H11	0.7014	0.5549	0.7504	0.075*	
C12	0.8989 (2)	0.7414 (6)	0.7402 (4)	0.1158 (13)	
C13A	0.8921 (5)	0.7427 (14)	0.6299 (6)	0.1189 (13)	0.455 (4)
H13A	0.8776	0.6568	0.6064	0.178*	0.455 (4)
H13B	0.9331	0.7626	0.6065	0.178*	0.455 (4)
H13C	0.8614	0.8096	0.6076	0.178*	0.455 (4)
C13B	0.9136 (5)	0.6293 (10)	0.6679 (7)	0.1189 (13)	0.545 (4)
H13D	0.9127	0.5441	0.6995	0.178*	0.545 (4)
H13E	0.9554	0.6436	0.6458	0.178*	0.545 (4)
H13F	0.8818	0.6309	0.6137	0.178*	0.545 (4)
C14A	0.9290 (6)	0.8780 (10)	0.7697 (9)	0.1189 (13)	0.455 (4)
H14A	0.8974	0.9470	0.7575	0.178*	0.455 (4)
H14B	0.9649	0.8951	0.7327	0.178*	0.455 (4)
H14C	0.9433	0.8768	0.8372	0.178*	0.455 (4)
C14B	0.8930 (5)	0.8728 (9)	0.6871 (8)	0.1189 (13)	0.545 (4)
H14D	0.8604	0.8658	0.6343	0.178*	0.545 (4)
H14E	0.9334	0.8945	0.6626	0.178*	0.545 (4)
H14F	0.8815	0.9416	0.7305	0.178*	0.545 (4)
C15A	0.9492 (5)	0.6380 (12)	0.7739 (9)	0.1189 (13)	0.455 (4)
H15A	0.9527	0.6333	0.8432	0.178*	0.455 (4)
H15B	0.9901	0.6630	0.7523	0.178*	0.455 (4)
H15C	0.9367	0.5523	0.7476	0.178*	0.455 (4)
C15B	0.9545 (4)	0.7453 (12)	0.8190 (6)	0.1189 (13)	0.545 (4)
H15D	0.9494	0.8209	0.8601	0.178*	0.545 (4)
H15E	0.9944	0.7526	0.7899	0.178*	0.545 (4)
H15F	0.9545	0.6648	0.8565	0.178*	0.545 (4)
C16	0.54110 (14)	0.1835 (3)	0.51971 (19)	0.0349 (7)	
C17	0.50240 (14)	0.2044 (3)	0.68156 (19)	0.0356 (7)	
C18	0.52587 (15)	0.3387 (3)	0.6835 (2)	0.0428 (7)	
H18	0.5225	0.3917	0.7378	0.051*	
C19	0.55269 (14)	0.3876 (3)	0.6071 (2)	0.0418 (7)	
H19	0.5658	0.4764	0.6086	0.050*	
C20	0.59296 (14)	0.3704 (3)	0.4448 (2)	0.0416 (7)	
H20A	0.5894	0.4669	0.4464	0.050*	
H20B	0.5706	0.3393	0.3848	0.050*	
C21	0.66279 (15)	0.3322 (3)	0.4470 (2)	0.0423 (7)	
C22	0.68630 (18)	0.2626 (3)	0.3729 (2)	0.0585 (9)	
H22	0.6582	0.2355	0.3210	0.070*	
C23	0.7508 (2)	0.2317 (4)	0.3736 (3)	0.0702 (11)	
H23	0.7649	0.1841	0.3220	0.084*	
C24	0.79479 (18)	0.2687 (4)	0.4475 (3)	0.0666 (10)	
C25	0.7703 (2)	0.3357 (5)	0.5223 (3)	0.0950 (15)	
H25	0.7980	0.3605	0.5752	0.114*	
C26	0.70607 (19)	0.3674 (5)	0.5218 (3)	0.0794 (13)	
H26	0.6919	0.4141	0.5738	0.095*	
C27	0.8656 (2)	0.2326 (5)	0.4482 (4)	0.1145 (6)	
C28A	0.8853 (5)	0.2512 (12)	0.3424 (7)	0.1154 (5)	0.458 (7)
H28A	0.8621	0.1884	0.3004	0.173*	0.458 (7)
H28B	0.9307	0.2362	0.3413	0.173*	0.458 (7)
H28C	0.8751	0.3404	0.3209	0.173*	0.458 (7)
C28B	0.8862 (5)	0.1729 (11)	0.3544 (7)	0.1154 (5)	0.542 (7)
H28D	0.8756	0.0796	0.3516	0.173*	0.542 (7)
H28E	0.9319	0.1834	0.3524	0.173*	0.542 (7)
H28F	0.8643	0.2180	0.3003	0.173*	0.542 (7)
C29A	0.9086 (5)	0.3213 (11)	0.5155 (8)	0.1154 (5)	0.458 (7)
H29A	0.8937	0.4119	0.5099	0.173*	0.458 (7)
H29B	0.9520	0.3163	0.4980	0.173*	0.458 (7)
H29C	0.9070	0.2915	0.5809	0.173*	0.458 (7)
C29B	0.9078 (5)	0.3574 (9)	0.4697 (8)	0.1154 (5)	0.542 (7)
H29D	0.8969	0.4244	0.4217	0.173*	0.542 (7)
H29E	0.9524	0.3337	0.4684	0.173*	0.542 (7)
H29F	0.9006	0.3917	0.5324	0.173*	0.542 (7)
C30A	0.8739 (5)	0.0849 (8)	0.4731 (8)	0.1154 (5)	0.542 (7)
H30A	0.8565	0.0672	0.5336	0.173*	0.542 (7)
H30B	0.9188	0.0625	0.4781	0.173*	0.542 (7)
H30C	0.8516	0.0319	0.4233	0.173*	0.542 (7)
C30B	0.8809 (5)	0.1323 (11)	0.5311 (8)	0.1154 (5)	0.458 (7)
H30D	0.8788	0.1768	0.5919	0.173*	0.458 (7)
H30E	0.9234	0.0969	0.5273	0.173*	0.458 (7)
H30F	0.8502	0.0608	0.5257	0.173*	0.458 (7)

### Atomic displacement parameters (Å^2^)

**Table:**

	*U*^11^	*U*^22^	*U*^33^	*U*^12^	*U*^13^	*U*^23^
O1	0.0835 (17)	0.0448 (13)	0.0513 (14)	0.0173 (12)	0.0200 (12)	0.0114 (11)
O2	0.113 (2)	0.0759 (18)	0.0492 (15)	0.0231 (16)	0.0290 (14)	0.0220 (13)
O3	0.0582 (13)	0.0391 (12)	0.0361 (11)	0.0011 (10)	0.0149 (10)	0.0008 (9)
O4	0.0661 (14)	0.0362 (11)	0.0408 (12)	−0.0098 (10)	0.0205 (10)	−0.0108 (9)
N1	0.0570 (16)	0.0341 (13)	0.0383 (14)	0.0070 (12)	0.0167 (11)	0.0010 (11)
N2	0.0482 (15)	0.0469 (15)	0.0373 (14)	0.0059 (12)	0.0135 (11)	−0.0004 (12)
N3	0.0477 (14)	0.0245 (12)	0.0349 (13)	−0.0023 (10)	0.0120 (11)	−0.0028 (10)
N4	0.0442 (14)	0.0264 (12)	0.0379 (13)	−0.0043 (10)	0.0091 (11)	−0.0016 (10)
C1	0.0461 (18)	0.0343 (16)	0.0377 (16)	−0.0001 (14)	0.0105 (13)	0.0009 (13)
C2	0.065 (2)	0.0451 (18)	0.0418 (18)	0.0046 (16)	0.0123 (15)	0.0103 (15)
C3	0.072 (2)	0.052 (2)	0.055 (2)	0.0218 (17)	0.0134 (17)	0.0121 (16)
C4	0.063 (2)	0.0496 (19)	0.053 (2)	0.0193 (17)	0.0166 (16)	−0.0026 (16)
C5	0.0477 (19)	0.074 (2)	0.0381 (17)	0.0025 (17)	0.0093 (14)	−0.0032 (16)
C6	0.0503 (19)	0.0573 (19)	0.0381 (16)	−0.0006 (16)	0.0106 (14)	−0.0018 (15)
C7	0.078 (3)	0.117 (3)	0.066 (2)	−0.027 (2)	0.029 (2)	−0.038 (2)
C8	0.079 (3)	0.126 (4)	0.083 (3)	−0.041 (3)	0.028 (2)	−0.030 (3)
C9	0.063 (2)	0.085 (3)	0.059 (2)	−0.007 (2)	0.0228 (18)	0.002 (2)
C10	0.074 (3)	0.094 (3)	0.058 (2)	−0.005 (2)	0.0288 (19)	−0.014 (2)
C11	0.063 (2)	0.074 (2)	0.051 (2)	−0.0068 (19)	0.0179 (17)	−0.0138 (18)
C12	0.083 (2)	0.149 (3)	0.122 (3)	−0.020 (2)	0.047 (2)	0.012 (3)
C13A	0.086 (2)	0.152 (3)	0.125 (3)	−0.020 (2)	0.046 (2)	0.012 (2)
C13B	0.086 (2)	0.152 (3)	0.125 (3)	−0.020 (2)	0.046 (2)	0.012 (2)
C14A	0.086 (2)	0.152 (3)	0.125 (3)	−0.020 (2)	0.046 (2)	0.012 (2)
C14B	0.086 (2)	0.152 (3)	0.125 (3)	−0.020 (2)	0.046 (2)	0.012 (2)
C15A	0.086 (2)	0.152 (3)	0.125 (3)	−0.020 (2)	0.046 (2)	0.012 (2)
C15B	0.086 (2)	0.152 (3)	0.125 (3)	−0.020 (2)	0.046 (2)	0.012 (2)
C16	0.0419 (17)	0.0282 (15)	0.0356 (16)	−0.0013 (12)	0.0082 (12)	−0.0019 (12)
C17	0.0418 (17)	0.0315 (15)	0.0338 (15)	0.0041 (13)	0.0041 (13)	0.0003 (12)
C18	0.056 (2)	0.0330 (16)	0.0403 (17)	−0.0023 (14)	0.0091 (14)	−0.0102 (13)
C19	0.0512 (19)	0.0276 (15)	0.0473 (18)	−0.0039 (13)	0.0082 (14)	−0.0082 (13)
C20	0.0535 (19)	0.0327 (15)	0.0403 (17)	−0.0048 (14)	0.0145 (14)	0.0030 (13)
C21	0.0511 (19)	0.0334 (16)	0.0440 (17)	−0.0083 (14)	0.0133 (15)	0.0022 (13)
C22	0.065 (2)	0.060 (2)	0.051 (2)	0.0038 (18)	0.0097 (17)	−0.0045 (17)
C23	0.076 (3)	0.062 (2)	0.077 (3)	0.010 (2)	0.032 (2)	−0.005 (2)
C24	0.053 (2)	0.059 (2)	0.090 (3)	−0.0041 (18)	0.023 (2)	0.000 (2)
C25	0.055 (3)	0.129 (4)	0.100 (3)	−0.010 (3)	0.001 (2)	−0.041 (3)
C26	0.056 (3)	0.105 (3)	0.078 (3)	−0.008 (2)	0.011 (2)	−0.042 (2)
C27	0.0693 (10)	0.1021 (11)	0.1757 (12)	0.0096 (10)	0.0308 (11)	0.0051 (11)
C28A	0.0702 (9)	0.1029 (10)	0.1765 (10)	0.0100 (9)	0.0305 (9)	0.0050 (10)
C28B	0.0702 (9)	0.1029 (10)	0.1765 (10)	0.0100 (9)	0.0305 (9)	0.0050 (10)
C29A	0.0702 (9)	0.1029 (10)	0.1765 (10)	0.0100 (9)	0.0305 (9)	0.0050 (10)
C29B	0.0702 (9)	0.1029 (10)	0.1765 (10)	0.0100 (9)	0.0305 (9)	0.0050 (10)
C30A	0.0702 (9)	0.1029 (10)	0.1765 (10)	0.0100 (9)	0.0305 (9)	0.0050 (10)
C30B	0.0702 (9)	0.1029 (10)	0.1765 (10)	0.0100 (9)	0.0305 (9)	0.0050 (10)

### Geometric parameters (Å, º)

**Table:**

O1—C1	1.211 (3)	C14B—H14E	0.9600
O2—C2	1.210 (4)	C14B—H14F	0.9600
O3—C17	1.231 (3)	C15A—H15A	0.9600
O4—C16	1.229 (3)	C15A—H15B	0.9600
N1—C1	1.370 (4)	C15A—H15C	0.9600
N1—C2	1.378 (4)	C15B—H15D	0.9600
N1—H1	0.8600	C15B—H15E	0.9600
N2—C4	1.365 (4)	C15B—H15F	0.9600
N2—C1	1.382 (4)	C17—C18	1.430 (4)
N2—C5	1.466 (4)	C18—C19	1.335 (4)
N3—C16	1.366 (3)	C18—H18	0.9300
N3—C17	1.386 (3)	C19—H19	0.9300
N3—H3	0.8600	C20—C21	1.503 (4)
N4—C19	1.370 (3)	C20—H20A	0.9700
N4—C16	1.373 (4)	C20—H20B	0.9700
N4—C20	1.469 (3)	C21—C26	1.363 (5)
C2—C3	1.440 (4)	C21—C22	1.369 (4)
C3—C4	1.328 (4)	C22—C23	1.380 (5)
C3—H3A	0.9300	C22—H22	0.9300
C4—H4	0.9300	C23—C24	1.367 (6)
C5—C6	1.503 (4)	C23—H23	0.9300
C5—H5A	0.9700	C24—C25	1.371 (5)
C5—H5B	0.9700	C24—C27	1.519 (6)
C6—C7	1.355 (5)	C25—C26	1.375 (6)
C6—C11	1.366 (4)	C25—H25	0.9300
C7—C8	1.380 (5)	C26—H26	0.9300
C7—H7	0.9300	C27—C29A	1.523 (8)
C8—C9	1.359 (5)	C27—C30A	1.525 (7)
C8—H8	0.9300	C27—C28B	1.529 (8)
C9—C10	1.371 (5)	C27—C30B	1.540 (8)
C9—C12	1.522 (6)	C27—C29B	1.543 (8)
C10—C11	1.384 (5)	C27—C28A	1.571 (8)
C10—H10	0.9300	C28A—H28A	0.9600
C11—H11	0.9300	C28A—H28B	0.9600
C12—C14B	1.508 (8)	C28A—H28C	0.9600
C12—C15A	1.519 (8)	C28B—H28D	0.9600
C12—C15B	1.524 (8)	C28B—H28E	0.9600
C12—C13A	1.527 (8)	C28B—H28F	0.9600
C12—C14A	1.545 (8)	C29A—H29A	0.9600
C12—C13B	1.554 (8)	C29A—H29B	0.9600
C13A—H13A	0.9600	C29A—H29C	0.9600
C13A—H13B	0.9600	C29B—H29D	0.9600
C13A—H13C	0.9600	C29B—H29E	0.9600
C13B—H13D	0.9600	C29B—H29F	0.9600
C13B—H13E	0.9600	C30A—H30A	0.9600
C13B—H13F	0.9600	C30A—H30B	0.9600
C14A—H14A	0.9600	C30A—H30C	0.9600
C14A—H14B	0.9600	C30B—H30D	0.9600
C14A—H14C	0.9600	C30B—H30E	0.9600
C14B—H14D	0.9600	C30B—H30F	0.9600
			
C1—N1—C2	128.2 (3)	C12—C15B—H15E	109.5
C1—N1—H1	115.9	H15D—C15B—H15E	109.5
C2—N1—H1	115.9	C12—C15B—H15F	109.5
C4—N2—C1	120.7 (2)	H15D—C15B—H15F	109.5
C4—N2—C5	120.2 (3)	H15E—C15B—H15F	109.5
C1—N2—C5	118.4 (3)	O4—C16—N3	122.2 (2)
C16—N3—C17	126.9 (2)	O4—C16—N4	122.1 (2)
C16—N3—H3	116.5	N3—C16—N4	115.7 (2)
C17—N3—H3	116.5	O3—C17—N3	119.8 (2)
C19—N4—C16	120.3 (2)	O3—C17—C18	126.2 (3)
C19—N4—C20	121.6 (2)	N3—C17—C18	113.9 (2)
C16—N4—C20	118.1 (2)	C19—C18—C17	119.8 (3)
O1—C1—N1	122.1 (3)	C19—C18—H18	120.1
O1—C1—N2	123.3 (3)	C17—C18—H18	120.1
N1—C1—N2	114.6 (3)	C18—C19—N4	123.3 (3)
O2—C2—N1	120.4 (3)	C18—C19—H19	118.4
O2—C2—C3	126.6 (3)	N4—C19—H19	118.4
N1—C2—C3	113.0 (3)	N4—C20—C21	112.1 (2)
C4—C3—C2	120.2 (3)	N4—C20—H20A	109.2
C4—C3—H3A	119.9	C21—C20—H20A	109.2
C2—C3—H3A	119.9	N4—C20—H20B	109.2
C3—C4—N2	123.3 (3)	C21—C20—H20B	109.2
C3—C4—H4	118.4	H20A—C20—H20B	107.9
N2—C4—H4	118.4	C26—C21—C22	116.7 (3)
N2—C5—C6	115.1 (3)	C26—C21—C20	121.8 (3)
N2—C5—H5A	108.5	C22—C21—C20	121.5 (3)
C6—C5—H5A	108.5	C21—C22—C23	121.4 (4)
N2—C5—H5B	108.5	C21—C22—H22	119.3
C6—C5—H5B	108.5	C23—C22—H22	119.3
H5A—C5—H5B	107.5	C24—C23—C22	122.4 (4)
C7—C6—C11	117.1 (3)	C24—C23—H23	118.8
C7—C6—C5	123.1 (3)	C22—C23—H23	118.8
C11—C6—C5	119.7 (3)	C23—C24—C25	115.5 (4)
C6—C7—C8	121.0 (4)	C23—C24—C27	121.9 (4)
C6—C7—H7	119.5	C25—C24—C27	122.5 (4)
C8—C7—H7	119.5	C24—C25—C26	122.4 (4)
C9—C8—C7	123.0 (4)	C24—C25—H25	118.8
C9—C8—H8	118.5	C26—C25—H25	118.8
C7—C8—H8	118.5	C21—C26—C25	121.6 (4)
C8—C9—C10	115.5 (4)	C21—C26—H26	119.2
C8—C9—C12	122.8 (4)	C25—C26—H26	119.2
C10—C9—C12	121.7 (4)	C24—C27—C29A	112.6 (6)
C9—C10—C11	122.0 (3)	C24—C27—C30A	108.9 (5)
C9—C10—H10	119.0	C29A—C27—C30A	112.2 (7)
C11—C10—H10	119.0	C24—C27—C28B	115.6 (6)
C6—C11—C10	121.3 (4)	C24—C27—C30B	107.5 (5)
C6—C11—H11	119.3	C28B—C27—C30B	109.1 (6)
C10—C11—H11	119.3	C24—C27—C29B	110.3 (5)
C14B—C12—C9	107.9 (5)	C28B—C27—C29B	106.5 (6)
C15A—C12—C9	111.0 (6)	C30B—C27—C29B	107.7 (7)
C14B—C12—C15B	110.7 (6)	C24—C27—C28A	107.3 (6)
C9—C12—C15B	109.9 (5)	C29A—C27—C28A	108.5 (6)
C9—C12—C13A	113.7 (6)	C30A—C27—C28A	107.1 (6)
C15A—C12—C14A	105.6 (6)	C27—C28A—H28A	109.5
C9—C12—C14A	112.5 (5)	C27—C28A—H28B	109.5
C13A—C12—C14A	105.0 (6)	C27—C28A—H28C	109.5
C14B—C12—C13B	109.0 (6)	C27—C28B—H28D	109.5
C9—C12—C13B	111.7 (5)	C27—C28B—H28E	109.5
C15B—C12—C13B	107.7 (6)	H28D—C28B—H28E	109.5
C12—C13A—H13A	109.5	C27—C28B—H28F	109.5
C12—C13A—H13B	109.5	H28D—C28B—H28F	109.5
C12—C13A—H13C	109.5	H28E—C28B—H28F	109.5
C12—C13B—H13D	109.5	C27—C29A—H29A	109.5
C12—C13B—H13E	109.5	C27—C29A—H29B	109.5
H13D—C13B—H13E	109.5	C27—C29A—H29C	109.5
C12—C13B—H13F	109.5	C27—C29B—H29D	109.5
H13D—C13B—H13F	109.5	C27—C29B—H29E	109.5
H13E—C13B—H13F	109.5	H29D—C29B—H29E	109.5
C12—C14A—H14A	109.5	C27—C29B—H29F	109.5
C12—C14A—H14B	109.5	H29D—C29B—H29F	109.5
C12—C14A—H14C	109.5	H29E—C29B—H29F	109.5
C12—C14B—H14D	109.5	C27—C30A—H30A	109.5
C12—C14B—H14E	109.5	C27—C30A—H30B	109.5
H14D—C14B—H14E	109.5	C27—C30A—H30C	109.5
C12—C14B—H14F	109.5	C27—C30B—H30D	109.5
H14D—C14B—H14F	109.5	C27—C30B—H30E	109.5
H14E—C14B—H14F	109.5	H30D—C30B—H30E	109.5
C12—C15A—H15A	109.5	C27—C30B—H30F	109.5
C12—C15A—H15B	109.5	H30D—C30B—H30F	109.5
C12—C15A—H15C	109.5	H30E—C30B—H30F	109.5
C12—C15B—H15D	109.5		

### Hydrogen-bond geometry (Å, º)

**Table:**

*D*—H···*A*	*D*—H	H···*A*	*D*···*A*	*D*—H···*A*
N1—H1···O3^i^	0.86	2.06	2.915 (3)	174
N3—H3···O4^ii^	0.86	2.03	2.851 (3)	160

Symmetry codes: (i) −*x*+1, −*y*+1, −*z*+2; (ii) −*x*+1, −*y*, −*z*+1.
